# Measurement force, speed, and postmortem time affect the ratio of CNS gray-to-white-matter elasticity

**DOI:** 10.1016/j.bpj.2025.03.009

**Published:** 2025-03-16

**Authors:** Julia Monika Becker, Alexander Kevin Winkel, Eva Kreysing, Kristian Franze

**Affiliations:** 1 Department of Physiology, Development and Neuroscience, University of Cambridge, Cambridge, Cambridgeshire, United Kingdom; 2 Institute of Medical Physics and Microtissue Engineering, Friedrich-Alexander-Universität Erlangen-Nürnberg, Erlangen, Bayern, Germany; 3 Max-Planck-Zentrum für Physik und Medizin, Erlangen, Bayern, Germany

## Abstract

For several decades, many attempts have been made to characterize the mechanical properties of gray and white matter, which constitute the two main compartments of the central nervous system, with various methods and contradictory results. In particular, the ratio of gray-to-white-matter elasticity is sometimes larger than 1 and sometimes smaller; the reason for this apparent discrepancy is currently unknown. Here, we exploited atomic force microscopy-based indentation measurements to systematically investigate how the measurement force, measurement speed, postmortem interval, and temperature affect the measured elasticity of spinal cord tissue and, in particular, the ratio of gray-to-white-matter elasticity (*K*_*g*_*/K*_*w*_). Within the explored parameter space, increasing measurement force and speed increased the measured elasticity of both gray and white matter. However, *K*_*g*_*/K*_*w*_ declined from values as high as ∼5 at low forces and speeds to ∼1 for high forces and speeds. *K*_*g*_*/K*_*w*_ also strongly depended on the anatomical plane in which the measurements were conducted and was considerably higher in transverse sections compared with longitudinal sections. Furthermore, the postmortem interval impacted both the absolute measured tissue elasticity and *K*_*g*_*/K*_*w*_. Gray matter elasticity started decreasing ∼3 h postmortem until reaching a plateau after ∼6 h. In contrast, white matter elasticity started declining from the beginning of the measurements until ∼6 h postmortem, when it also leveled off. As a result, *K*_*g*_*/K*_*w*_ increased until ∼6 h postmortem before stabilizing. Between 20 and 38°C, both gray and white matter elasticity decreased at a similar rate without affecting *K*_*g*_*/K*_*w*_. We have thus identified differences in the response of gray and white matter to varying strains and strain rates, and the postmortem interval, and excluded temperature as a factor affecting *K*_*g*_*/K*_*w*_. These differential responses likely contribute to the contradictory results obtained with different methods working in different strain regimes.

## Significance

We here showed that the mechanical response of CNS gray and white matter to an applied force differentially depends on measurement parameters such as the speed and magnitude of the applied forces, the postmortem interval, and the anatomical axis along which measurements are conducted. These results broaden our understanding of CNS mechanics and pave the way for better and more targeted experimental design of future experiments. Ultimately, they may help to reconcile seemingly contradictory results in the literature concerning the ratio of gray-to-white-matter elasticity.

## Introduction

The mechanical properties of central nervous system (CNS) tissue are critical for regulating diverse neuronal and glial cell functions (1). Tissue stiffness, for example, contributes to developmental processes, such as axon path finding (2,3) and brain folding (4), to tissue maintenance during adult neurogenesis (5), as well as to pathological processes, such as brain tumor chemoresistance (6) or axonal remyelination (7). Knowledge about mechanical CNS tissue properties, which are highly dynamic and change during development, aging, and pathological processes, is therefore important for our understanding of normal and dysregulated CNS cell function.

For over half a century, various methods have been exploited to measure CNS mechanics in health and disease, resulting in differing and sometimes even contradictory findings. In particular, there is still no consensus regarding mechanical differences between gray matter, which mostly contains neuronal cell bodies and synapses, and white matter, which is predominantly built up by myelinated axon tracts. The ratio of gray-to-white-matter tissue elasticity (*K*_*g*_*/K*_*w*_) is not only important for cortical folding (4) but also in the context of mechanical trauma, as the relative mechanical properties of gray and white matter affect the stress and strain patterns resulting from traumatic deformations (8). Yet, in some reports, gray matter is stiffer than white matter (9,10,11,12,13), while in others white matter is stiffer than gray matter (14,15,16,17), and there are publications where both tissue types are mechanically similar (18,19). These findings somewhat cluster according to the methodologies used to measure tissue mechanics. Studies employing atomic force microscopy (AFM) (10,11,12,13), which applied forces in the ∼nN regime at a frequency on the order of ∼1 Hz, usually find *K*_*g*_*/K*_*w*_ > 1, whereas studies using nanoindentation testing (14,15,16) or magnetic resonance elastography (MRE) (17), which apply comparatively larger forces (approximately hundreds of *μ*N) or forces at higher frequencies (∼50–100 Hz), respectively, commonly find *K*_*g*_*/K*_*w*_ < 1.

Although it is known that many parameters influence overall CNS tissue elasticity, such as the applied strain amplitude (i.e., the amount of deformation) (20,21), the strain rate (i.e., the speed of deformation) (10,15), the “freshness” of the sample (i.e., the postmortem time) (22,23), and the temperature (24,25), their effect on the gray-to-white-matter elasticity ratio *K*_*g*_*/K*_*w*_ has not yet been systematically investigated.

Here, we used AFM-based indentation tests to determine the reduced apparent elastic modulus K=E1−ν2 (a measure of elasticity, with *E* being the Young’s modulus and *v* the Poisson’s ratio) of fresh rat spinal cord sections, which are characterized by clearly defined gray and white matter areas. We conducted representative elasticity measurements across entire sections, systematically varying the measurement force (i.e., strain), measurement speed (i.e., strain rate), anatomical plane (i.e., directionality of the force application relative to the orientation of the tissue), postmortem interval, and measurement temperature. We found that, while temperature did not affect the gray-to-white-matter elasticity ratio *K*_*g*_*/K*_*w*_, force, speed, anatomical plane, and the postmortem interval did.

## Materials and methods

For ease of reading, general protocols are presented first, followed by details and deviations for individual experiments. All reagents were purchased from Sigma-Aldrich (St. Louis, MO, USA), unless stated otherwise.

### Artificial cerebrospinal fluid solutions

Artificial cerebrospinal fluid (aCSF) solutions established for electrophysiology (26) were used to ensure optimal tissue quality and prepared fresh every day. Slicing aCSF contained 5 mM ethyl pyruvate, 26 mM choline bicarbonate, 2 mM NaOH (Fisher Scientific, Loughborough, UK), 2 mM kynurenic acid, 191 mM sucrose, 20 mM glucose, 1 mM (+)-sodium L-ascorbate, 3 mM myo-inositol, 0.75 mM potassium gluconate, 1.25 mM KH_2_PO_4_, 4 mM MgSO_4_, and 1 mM CaCl_2_ (Fluka^TM^, Muskegon, MI, USA) in autoclaved ddH_2_O. Measuring aCSF contained 5 mM ethyl pyruvate, 15 mM glucose, 1 mM (+)-sodium L-ascorbate, 3 mM myo-inositol, 121 mM NaCl, 3 mM KCl, 1.25 mM NaH_2_PO_4_, 25 mM NaHCO_3_, 1.1 mM MgCl_2_, and 2.2 mM CaCl_2_ (Fluka, Muskegon, MI, USA) in autoclaved ddH_2_O. Both aCSF solutions were bubbled with carbogen (95% O_2_/5% CO_2_) for at least 30 min before and throughout the experiments. Slicing aCSF was kept on ice before and during use, measuring aCSF was kept at room temperature. The resulting pH was 7.20–7.33 for the slicing aCSF and 7.34 for the measuring aCSF.

### Animals and sample preparation

Details about the rats used in this study are shown in Table 1. To reduce animal numbers, spare rats from other research groups were used when available; otherwise, rats were purchased from Charles River and Envigo. All work involving animals was conducted in keeping with the Animals (Scientific Procedures) Act 1986 and approved by the relevant authorities according to the University of Cambridge Animal Welfare and Ethical Review Body (AWERB) processes.

**Table 1:** Animals used for experiments

Experiment	Strain	Sex and numbers	Median weight (range) (g)	Estimated age (weeks)	Sectioning plane and vertebral level
Force and speed	Lister Hooded	female (*N* = 13); male (*N* = 9)	219 (165–283)	6–10	transverse (C5); sagittal (C4–C6); horizontal (C4–C6)
Postmortem interval	Wistar; Wistar Han	female (*N* = 11)	330 (282–509)	46–49	transverse (C5)
Temperature	Lister Hooded	female (*N* = 6)	215 (200–226)	9–10	transverse (C5)

Rats were euthanized with an overdose of pentobarbital (2000 mg/kg i.p.) under isoflurane anesthesia. Typical times required for individual steps of the dissection procedure relative to the time of death are indicated in brackets. A thoracotomy was performed and a shortened intravenous catheter was placed through the left atrium into the proximal aorta and stitched in place (∼3 min), and immediate perfusion with ice-cold slicing aCSF at 15–16 mL/min was started. A dorsal midline incision was made and the cervical spinal cord of the vertebral segments C4 to C6 was exposed via a laminectomy and gently removed from the body (∼18 min). Dissection was continued in a perfused dish (5 mL/min) on ice and the dura mater and leptomeninx were carefully removed (∼35 min). Samples were embedded in 4% low gelling temperature agarose in PBS and sectioned on a VT1000S vibratome (Leica, Nussloch, Germany) in slicing aCSF on ice with a Gillette Platinum blade (Procter & Gamble, Schwalbach am Taunus, Germany). Section thickness was set to 999 *μ*m, forward speed to 50 *μ*m/s, vibration frequency to 60 Hz, and vibration amplitude to 1 mm. Sections were immediately transferred to bubbled measuring aCSF at room temperature (∼50–75 min). When sectioning the horizontal or sagittal planes, care was taken to select tissue sections for AFM measurements where the location of the gray-to-white-matter boundary did not change much through the thickness of the section. The section chosen for AFM was transferred to a tissue culture dish (Ø 40 mm, TPP), held in place by small dots of cyanoacrylate glue between the dish and the agarose surrounding the sample, and submerged in measuring aCSF.

### AFM setup and measurements

Force-distance curves were acquired with our custom-built AFM setup in spectroscopy mode. The setup consists of a CellHesion 200 AFM head on a motorized precision stage with a Vortis Advanced Control Station (all JPK, Berlin, Germany) and an AxioZoom.V16 upright microscope (Zeiss, Jena, Germany) with an Zyla sCMOS camera (Andor Technology, Belfast, UK), mounted on an air table (RS 2000, Newport, Irvine, CA, USA). The dish with the tissue sample was placed in a PetriDishHeater (JPK, Berlin, Germany) set to 34°C, which resulted in a temperature of ∼32.5°C near the sample. To assess the effect of temperature, the samples were instead placed inside a BioCell (JPK, Berlin, Germany), using setpoint temperatures of 20, 24.5, 29, 33.5, and 38°C in either ascending or descending order (*N* = 3 animals each). Samples were given sufficient time (∼15 min) to equilibrate at each new temperature level before measurements were started. In all cases, bubbled measuring aCSF was continuously exchanged with a perfusion pump throughout the experiment at a rate of 0.72 mL/min.

For the majority of experiments, we used Arrow TL1 tipless silicon cantilevers (NanoWorld, Neuchâtel, Switzerland) with a spring constant of 0.083–0.093 N/m. For experiments investigating the effect of force and speed, an Arrow TL1 cantilever with a spring constant of 0.374 N/m (*N* = 2 animals) and TL-FM cantilevers (Nanosensors, Neuchâtel, Switzerland) with spring constants of 1.849–2.757 N/m (*N* = 20 animals) were used instead to allow for higher indentation forces. Cantilever spring constant was determined contact-free in air with the in-built thermal noise method of the JPK SPMControl software (JPK, Berlin, Germany). Spherical polystyrene beads of 44.65 *μ*m radius (microParticles, Berlin, Germany) were glued to the cantilever tip using M-Bond 610 (Micro-Measurements, Wendell, NC, USA). Cantilever sensitivity was determined on a glass slide in buffer before each experiment. AFM measurements were conducted at a constant indentation speed of 20 *μ*m/s in closed loop with a force of 30 nN at a sample rate of 1000 Hz. Experiments investigating the effect of force and speed were conducted with varying combinations of indentation forces of 30, 90, 150, 300, 600, 1200, and 1500 nN and indentation speeds of 20, 100, 400, 800, and 1200 *μ*m/s in closed loop, and sample rates were chosen to ensure a minimum of 2500 points on the extend part of the recorded force-distance curve. AFM measurement grids containing many individual measurement points, also called “maps,” were tailored to each sample’s geometry using a custom-written MATLAB (The MathWorks, Natick, MA, USA) routine to cover the sample in a representative way. Generally, maps were measured in mediolateral direction; to investigate the effect of force and speed, various measurement directions were used. Table 2 contains the start and end time of AFM measurements for individual experiments and the grid resolutions used. To examine the effect of postmortem time and temperature, the same map was repeatedly remeasured, either after a certain amount of time had elapsed or after sample temperature had been changed, respectively. In the former case, maps were repeatedly measured every ∼30 or ∼60 min. Maps which covered both halves of the spinal cord took ∼90 min to complete.

**Table 2:** Start and end time and resolution of AFM measurements

Experiment	Measurement start (h:min postmortem)	Measurement end (h:min postmortem)	Grid resolution (*μ*m)
Force and speed	1:30–2:30	3:05–8:35	50–200
Postmortem interval	1:30–2:04	6:27–11:06	178–260
Temperature	2:10–2:48	5:43–6:11	182–201

### AFM data analysis

All AFM data analysis was carried out with MATLAB (The MathWorks ). A custom-written script (10) was used to determine the reduced apparent elastic modulus *K* from each force-distance curve after determining the contact point with a fitting algorithm. All elasticity values are reported as *K* rather than the Young’s modulus *E* to avoid assumptions about the value of Poisson’s ratio ν ($K=E/\left(1-\nu^{2}\right)$). To obtain *K*, the Hertz model $F=\frac{4\;}{3}K\;\sqrt[{2}]{R{\;\delta }^{3}\;}$ (27) was fitted to the curve data (*R* is bead radius; *F* is exerted force; *δ* is indentation depth) using a custom-written script. The use of the Hertz model for a paraboloid indenter was considered appropriate for the analysis of the postmortem time and temperature data acquired with a spherical indenter, as more than two-thirds of all measurements satisfied δR<13, where both indenter geometries are similar. For data analysis concerning the effect of force and speed, the in-built Sneddon model for a spherical indenter (28,29) from the JPK Data Processing Software (JPK, Berlin, Germany) was used instead to provide greater accuracy for higher indentation depths. Using an overview image of each sample with an overlay of the measurement grid, data points were manually segmented into white and gray matter. Custom-written software for AFM and AFM data analysis is available from https://github.com/FranzeLab/AFM-data-analysis-and-processing/tree/Batchforce_1.1/Batchforce and https://github.com/FranzeLab/AFM-data-analysis-and-processing/tree/master/JuliaBeckerThesis. All raw data force-indentation curves used for analysis are available online (Zenodo: https://doi.org/10.5281/zenodo.14630529).

To obtain the stiffness *k* for comparison with the apparent reduced elastic modulus *K*, a slope was fitted to the force-indentation data in the range of 90–100% exerted force using the JPK Data Processing Software (JPK, Berlin, Germany).

### Statistical and regression analysis

Statistical analysis was carried out with Prism 9 or 10 for macOS (GraphPad Software, Boston, MA, USA). The significance level was α=0.05. To assess whether AFM measurements themselves altered the tissue’s mechanical properties, a two-tailed one-sample *t*-test against a hypothetical value of 1 was used. To assess the effect of directionality, ANOVA was used to compare *K* values and the *K*_*g*_*/K*_*w*_ ratio obtained in different anatomical planes with the same parameter combinations for force and speed, followed by Tukey’s multiple comparisons test for individual group comparisons. For this analysis, only parameter combinations were assessed for which data was available for all three planes and at least three animals per plane. To assess the effect of repeated AFM measurements, a two-tailed unpaired *t*-test was used on log-transformed data. For regression analysis concerning the effect of temperature, data points from all maps of all animals were pooled. Each data point constituted the median apparent elastic modulus over the median temperature of one elasticity map in one animal. For the *K*_*g*_*/K*_*w*_ ratio, the ratio of the median gray to the median white matter apparent elastic modulus over the mean of the median gray and white matter temperature was used. An extra-sum-of-squares F-test was used to compare a linear regression model to a horizontal line. To investigate the relationship between the apparent reduced elastic modulus *K* and the stiffness *k*, correlation analysis and linear regression was conducted with MATLAB (The MathWorks).

## Results

To minimize cell damage and maximize sample viability, care was taken to dissect the tissue as carefully and quickly as possible, while constantly supplying it with oxygenated buffer solutions optimized for rodent CNS tissue (26) at specific temperatures (for details, see materials and methods). AFM measurements started at approximately 1.5–2 h postmortem. Throughout the study, we used AFM cantilevers with spherical beads of 44.65 *μ*m radius glued to their tips to exert forces on spinal cord tissue sections.

### Force and speed of AFM measurements strongly affect the measured tissue elasticity and *K*_g_/*K*_w_

**Figure 1:**
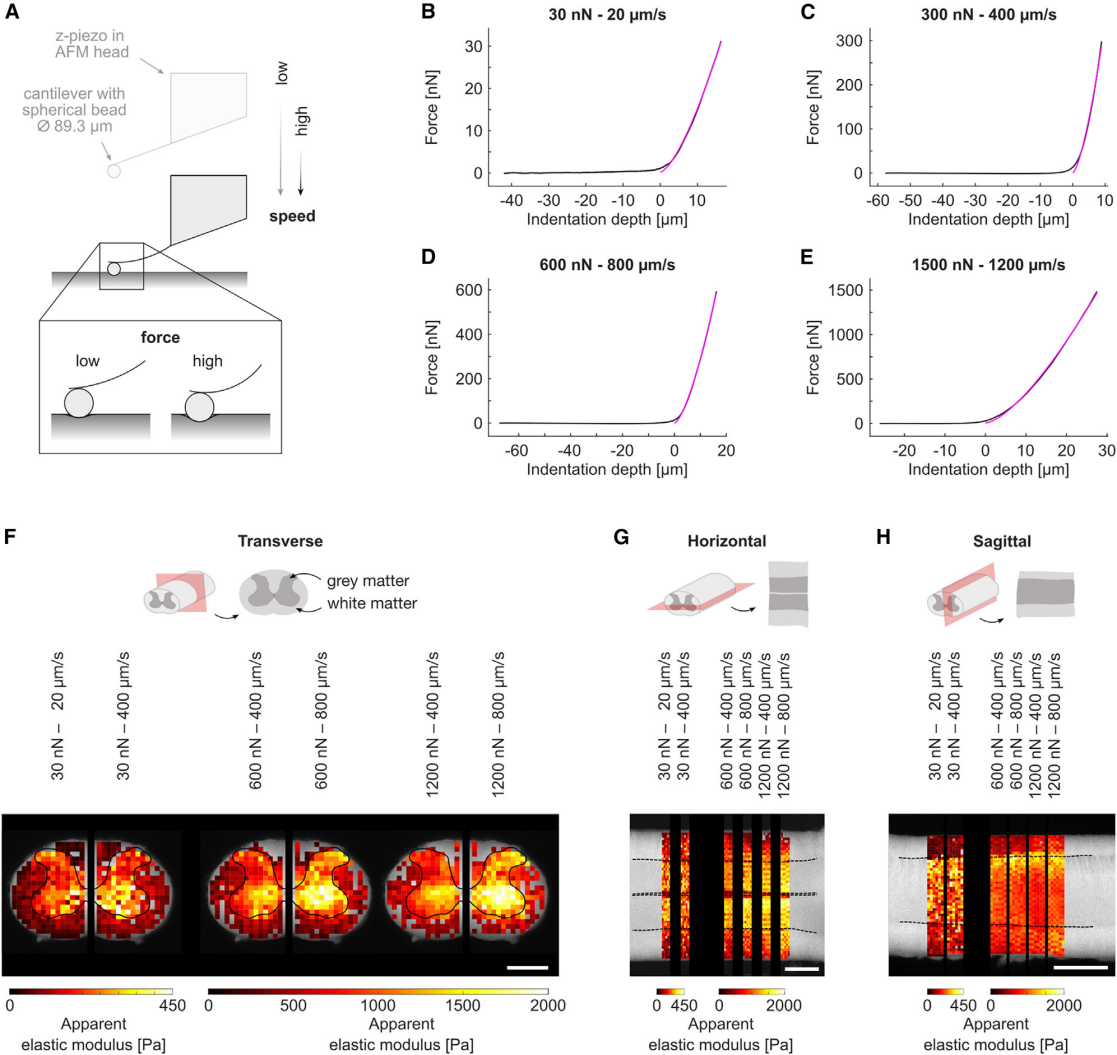
Choice of AFM measurement parameters force and speed affects the apparent reduced elastic modulus *K*. (*A*) Schematic of atomic force microscopy (AFM) setup. (*B–E*) Example force-distance curves (*black*) obtained from AFM measurements with different parameter combinations. The Sneddon model (*pink*) was fitted to the extend parts of the force-distance curves to obtain the respective apparent reduced elastic modulus *K*. (*F–H*) Schematics of the (*F*) transverse, (*G*) horizontal, and (*H*) sagittal planes (gray matter inside: *dark gray*; white matter outside: *light gray*; anatomical plane: *red*). For each anatomical plane, six AFM elasticity heatmaps are shown, which were acquired on the same example tissue section with different combinations of measurement forces and speeds. Different parameter combinations yield different apparent reduced elastic moduli on the same tissue section. Elasticity heatmaps are overlaid on a bright-field image of the measured tissue section. Images are broken for better visual segregation of the different parameter combinations. Each pixel represents one measurement. *Dashed black lines*: gray-to-white-matter boundary. Scale bars, 1000 *μ*m.

AFM permits spatially resolved mechanical probing of a tissue section. Force-distance curves obtained during an experiment can be fitted with the Sneddon or Hertz models (see materials and methods) to obtain the measured tissue elasticity, i.e., the reduced apparent elastic modulus *K*. AFM also allows for systematic variation of key measurement parameters, i.e., force and speed, which affect the measured *K* values (Fig. 1).

**Figure 2:**
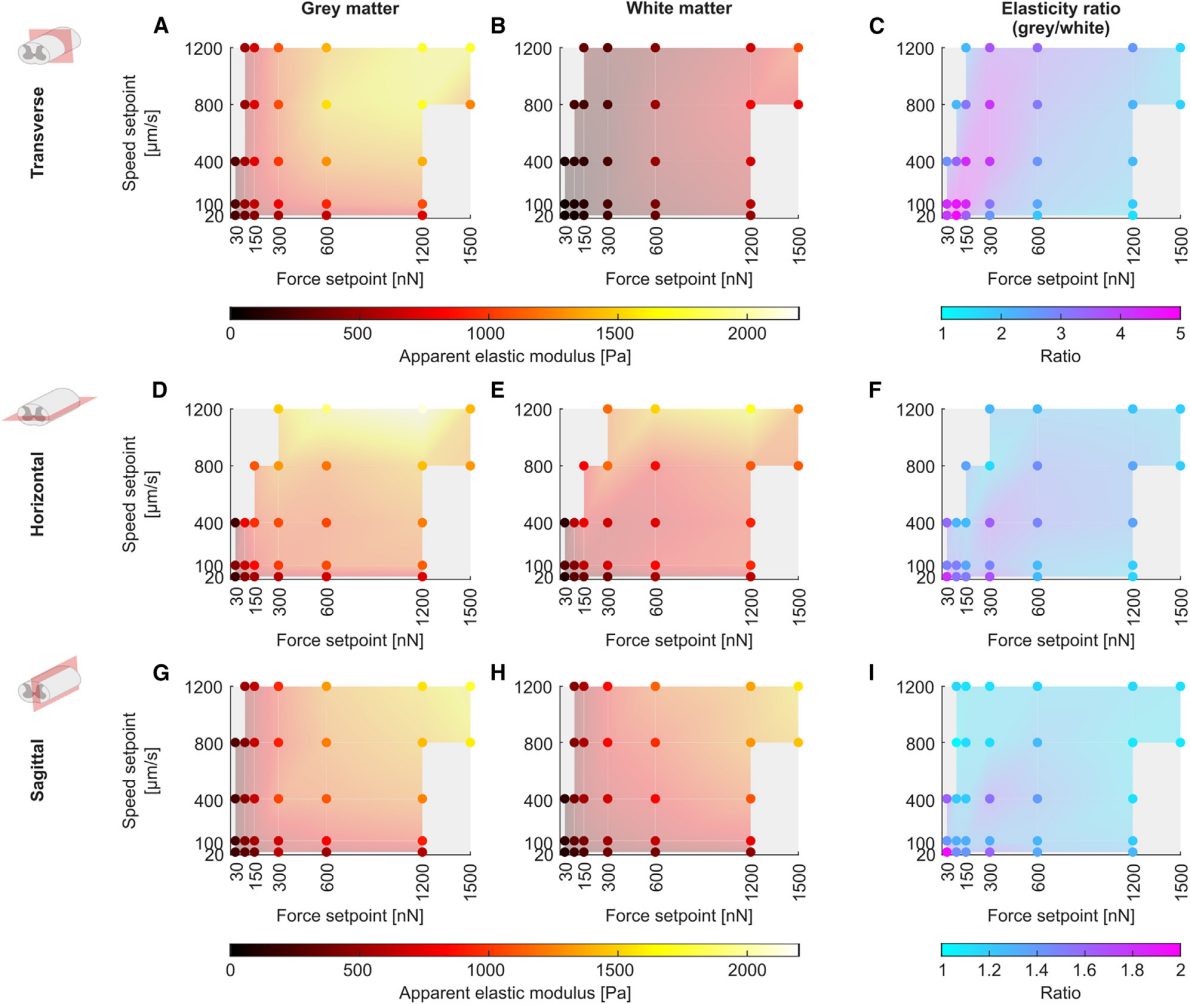
Increasing force and speed of AFM measurements increases the measured elasticity of gray and white matter but decreases the ratio of gray-to-white-matter elasticity. Apparent elastic moduli *K* for (*A*, *D*, and *G*) gray and (*B*, *E*, and *H*) white matter as well as (*C*, *F*, and *I*) gray-to-white-matter elasticity ratios *K*_*g*_*/K*_*w*_ for (*A–C*) transverse, (*D–F*) horizontal, and (*G–I*) sagittal sections. The mean of all animals’ median *K* and the *K*_*g*_*/K*_*w*_ ratios (i.e., median gray matter elasticity/median white matter elasticity) are represented by colored dots; the color represents the apparent elastic modulus or the ratio, respectively, as shown in the color bars. The color of the shaded areas between data points is interpolated for better visualization. While the elasticity of both gray and white matter increases with increasing force and measurement speed, their ratio *K*_*g*_*/K*_*w*_ drops. The number of animals (*N*) and AFM measurements (*n*) as well as the individual median gray and white matter elasticity values and *K*_*g*_*/K*_*w*_ ratio of each animal, respectively, are provided in Tables S1 and S2. In total, 27,940 measurements contributed to this data set. For the projection of these plots showing measured elasticity versus force or measured elasticity versus speed, see Figs. S3 and S4, respectively.

To assess how force and speed affect *K*, which effectively is a measure of tissue stiffness, in different anatomical planes, we conducted AFM indentation measurements on transverse, horizontal, and sagittal spinal cord tissue sections and systematically varied both measurement force *F* and measurement speed *s* within the limits dictated by the setup’s capabilities over two orders of magnitude (*F* = 30–1500 nN and *s* = 20–1200 *μ*m/s) (Fig. 2).

First, we confirmed that the AFM setup accurately achieves the setpoint forces and speeds over this range (Fig. S1). Measurements that deviated from the target values by more than 10% (e.g., due to exceeding the AFM’s z-movement range and thus not reaching the force setpoint) were excluded. Next, we assessed whether AFM measurement themselves altered mechanical tissue properties if conducted with the forces and speeds in this range. We took repeated measurements at the same location and compared the resulting tissue elasticity from consecutive measurements. As the *K* values did not differ to a biologically relevant degree (median value changes by −2, +4, and +5% in the transverse, horizontal, and sagittal plane, respectively; all three planes: *p* < 0.0001) (Fig. S2), we averaged data from repeated measurements for further analysis.

We found that, in both tissue compartments (i.e., gray and white matter) in all sectioning planes, except in transversely cut white matter, an increase in either force or speed generally led to an increase in the measured apparent elastic modulus. Increases at the lower end of the examined parameter space for both force and speed, from 30 to ∼300 nN and from 20 to 100 *μ*m/s, had a comparably greater effect than further increases of either parameter, indicating a nonlinear viscoelastic response (Figs. 2, S3, and S4). In transversely cut white matter, however, speed changes had little effect on *K* (Figs. 2 *B*, S3 *B*, and S4 *B*).

**Figure 3:**
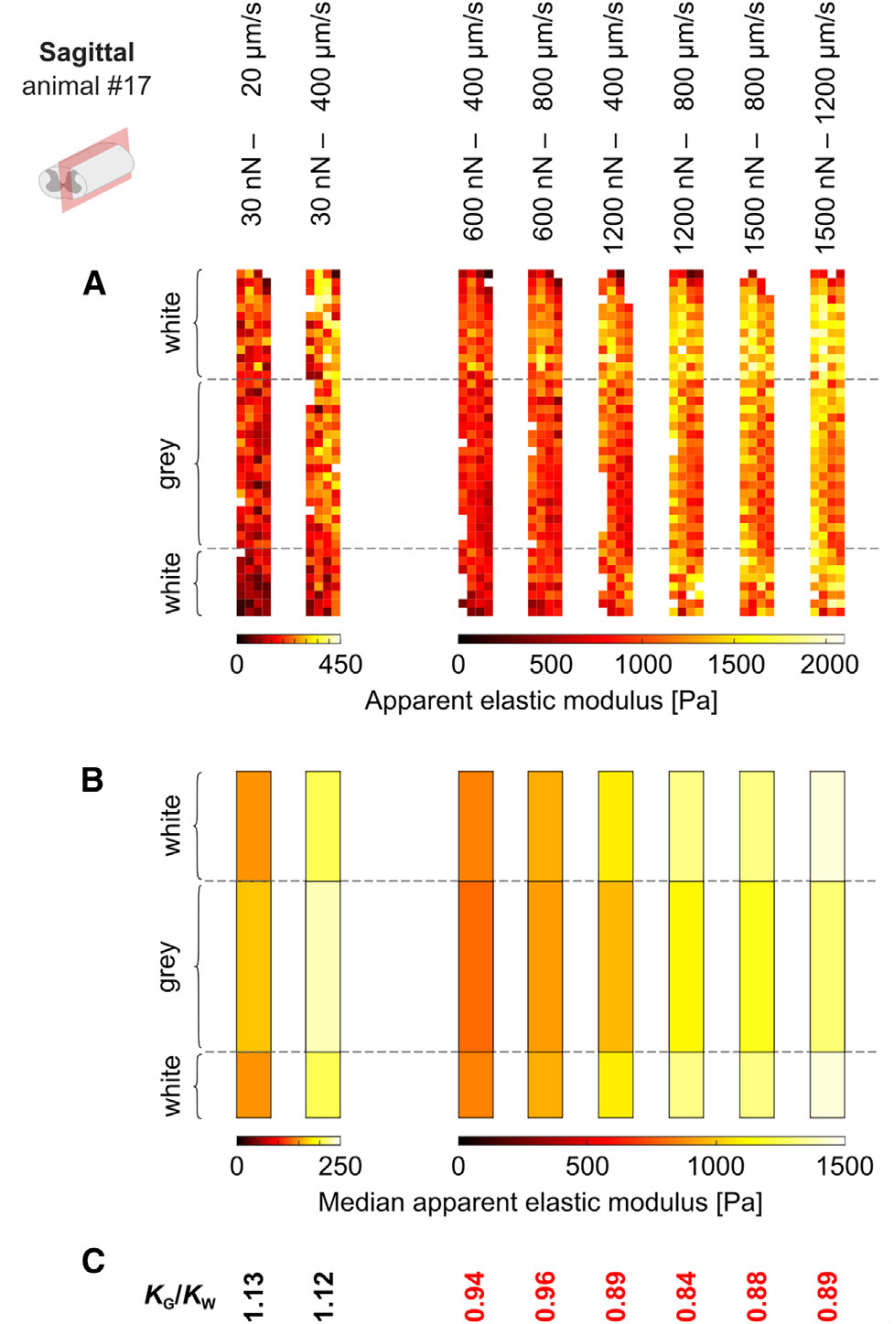
*K*_*g*_*/K*_*w*_ ratio can fall below 1 at high forces and speeds. In one animal (#17) measured in the sagittal plane, *K*_*g*_*/K*_w_ ratios were <1 for forces of ≥600 nN at speeds ≥400 *μ*m/s. (*A*) Individual AFM measurements and (*B*) median gray and white matter *K* values for all parameter combinations tested in this animal. Parameter combinations are annotated at the top of the figure and relate to all panels. (*A*) Each measurement or (*B*) median gray and white matter *K* values are represented by a color-coded pixel or rectangle, respectively, illustrating the local apparent elastic modulus as shown in the color bars. For clarity, data acquired with 30 nN force are displayed with separate color bars. *Dashed black lines*: gray-to-white-matter boundary. (*C*) *K*_*g*_*/K*_*w*_ ratio in this animal for every parameter combination. Ratios below 1 are indicated in red.

In all anatomical planes, gray and white matter were differentially affected by the tested measurement parameters. As force and speed were increased, gray matter stiffened relatively less than white matter, and thus the gray-to-white-matter elasticity ratio *K*_*g*_*/K*_*w*_ predominantly decreased with increasing force and speed (Fig. 2, *C*, *F*, and *I*). All mean *K*_*g*_*/K*_*w*_ ratios were greater than 1, indicating that, across the anatomical planes and parameter combinations tested here, gray matter was stiffer than white matter. However, the smallest mean *K*_*g*_*/K*_*w*_ ratio was 1.02 in the sagittal plane, thus approaching 1 and indicating that the measured gray and white matter tissue elasticity were nearly identical. In the sagittal plane, the *K*_*g*_*/K*_*w*_ ratio fell below 1 in a single animal, when forces of ≥600 nN were applied at speeds of ≥400 *μ*m/s, indicating that the measured tissue elasticity of white matter exceeded that of gray matter with these parameter combinations (Fig. 3; Tables S1 and S2).

### *K*_g_/*K*_w_ differs across anatomical planes and is highest in transverse sections

While gray matter elasticity was not significantly different across the three anatomical planes (Fig. S5), we found anisotropy in white matter elasticity for certain parameter combinations (Fig. S6). Despite not being statistically significant in all cases, white matter was consistently softest in the transverse plane (where axon tracts were cut predominantly perpendicular to their long axis and likely relaxed) and stiffer in the horizontal and sagittal planes (where tissue was cut parallel to most axon tracts). Gray matter, on the other hand, displayed a general but not statistically significant trend to be stiffest in the transverse plane. As a result, *K*_*g*_*/K*_*w*_ was significantly higher in the transverse plane compared with either of the other two longitudinal planes for all parameter combinations assessed. Between the horizontal and the sagittal planes, *K*_*g*_*/K*_*w*_ did not differ significantly (Fig. S7).

### Tissue elasticity decreases with time after death, whereas *K*_g_/*K*_w_ increases and then plateaus

From here, all experiments were conducted on transverse spinal cord sections with a setpoint force of 30 nN at a speed of 20 *μ*m/s. To investigate the effect of postmortem time on tissue elasticity, we conducted AFM measurements between 1:30 and 11 h postmortem (Table 2). Time points before 1:30 h postmortem could not be assessed due to the time required for the complex sample preparation and AFM setup procedure.

We repeatedly measured the same region of interest every 30–60 min at ∼32.5°C. We confirmed that multiple repeated AFM measurements did not alter the mechanical properties of the tissue by exploiting the mirror-symmetrical structure and mechanics of the spinal cord across the midline. We compared the elasticity changes occurring in one half of a spinal cord section which had been continuously remeasured (seven times in total) with those in the other half of the same section where measurements were conducted only once at the start and once at the end of the experiment and confirmed that the changes in both halves were not significantly different (Fig. S8).

**Figure 4:**
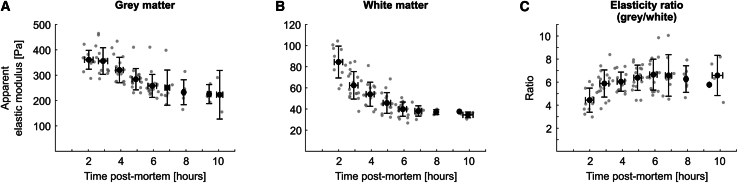
Gray and white matter tissue elasticity decline with time after death, whereas gray-to-white-matter elasticity ratio increases. Transverse spinal cord sections of 11 rats were used to record AFM maps 6–13 times every ∼30–60 min. Data are shown separately for (*A*) gray matter, (*B*) white matter, and (*C*) the ratio of gray-to-white-matter elasticity. While gray matter elasticity only started dropping after ∼3 h postmortem, white matter elasticity declined from the beginning. Both tissue compartments reached a plateau after ∼6 h postmortem. As white matter elasticity declined faster than gray matter elasticity, *K*_*g*_*/K*_*w*_ increased until ∼6 h postmortem. *Gray dots*: (*A* and *B*) median *K* over median time postmortem per map (gray matter: 28–56 measurements/map/animal, in total 3591 measurements; white matter: 36–95 measurements/map/animal, in total 5300 measurements) or (*C*) the ratio of median gray to median white matter elasticity over the mean of the median times postmortem at which the map was acquired. *Black dots* with error bars: mean ± SD for one-hour bins, starting at 1:30 h postmortem, of data shown in gray. Data were acquired with 30 nN force at 20 *μ*m/s speed. Maps which were not completed in time were excluded from data analysis, resulting in 6–11 usable maps per animal.

Gray matter tissue elasticity was initially relatively stable until ∼3 h postmortem. After ∼3 h, it started declining until ∼6 h postmortem, when gray matter elasticity reached another plateau of approximately two-thirds of its starting values. In the white matter, by contrast, a pronounced elasticity decrease was apparent immediately from the start of the observation period until ∼6 h postmortem, before white matter tissue elasticity plateaued at approximately 50% of its starting values. As a result, the gray-to-white-matter elasticity ratio *K*_*g*_*/K*_*w*_ increased slightly until ∼6 h postmortem before it leveled out (Fig. 4). However, the dynamics of the *K*_*g*_*/K*_*w*_ ratio were overall relatively variable across different animals.

### Temperature affects absolute tissue elasticity but not *K*_g_/*K*_w_

**Figure 5:**
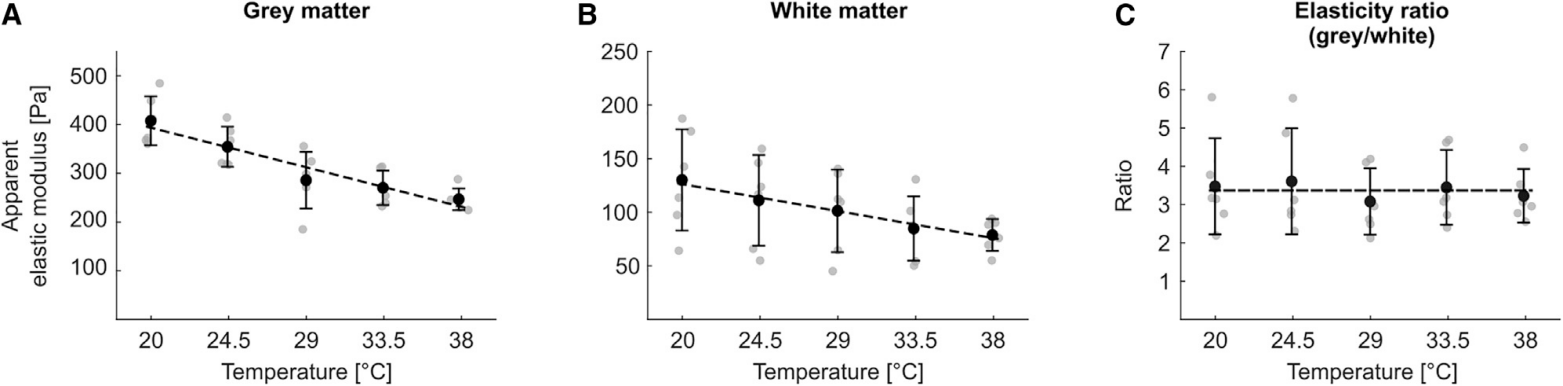
Gray and white matter tissue elasticity decrease with increasing temperature while the ratio of gray/white matter elasticity is unaffected. AFM maps were repeatedly recorded at five temperature levels (20, 24.5, 29, 33.5, and 38°C). Data are shown for (*A*) gray matter, (*B*) white matter, and (*C*) the ratio of gray-to-white-matter elasticity. *Gray dots*: (*A* and *B*) median *K* per animal over median measured temperature (gray matter: 35–48 measurements/temperature level/animal, in total 1308 measurements; white matter: 44–62 measurements/temperature level/animal, in total 1680 measurements) or (*C*) the ratio of median gray to median white matter elasticity per animal over the mean of the median measured temperatures for gray and white matter. *Black dots* with error bars: mean ± SD of data shown in gray, binned for each temperature level. The SD along the temperature axis was very small (<0.33°C) and is not shown for clarity. *Black dashed lines*: (*A* and *B*) linear regressions; (*C*) horizontal line. The gray-to-white-matter elasticity ratio is not dependent on temperature. Regression analysis details are provided in Table 3. Data were acquired with 30 nN force at 20 *μ*m/s speed.

Finally, we investigated the effect of temperature on spinal cord tissue elasticity in the range of 38–20°C, representing physiological rat body temperature and typical room temperature, respectively. Gray and white matter tissue elasticity both increased as temperature decreased (Fig. 5, *A* and *B*; Table 3). As gray and white matter elasticity changed at an almost identical rate (gray matter: 3.9% per 1°C; white matter: 3.7% per 1°C; see Table 3), *K*_*g*_*/K*_*w*_ was very stable in the examined temperature window and independent of the temperature (Fig. 5 *C*).

**Table 3:** Regression analysis of tissue elasticity *K* (Pa) as a function of temperature *x* (°C)

**Choice of model**	Gray matter	White matter	Elasticity ratio (gray-to-white)
Null hypothesis	In all cases: Horizontal line (*K = c*)		
Alternative hypothesis	In all cases: Linear model (*K = a^∗^x + b*)		
Preferred model (*p* value)	Linear model (*p* < 0.0001)	Linear model (*p* = 0.01)	Horizontal line (*p* = 0.59)

**Model parameters**			

*a* (slope) (Pa/°C)	−9.0	−2.8	–
*b* (intercept) (Pa)	573.5	182.2	–
*c* (constant = mean of values)	–	–	3.369
Adjusted r^2^	0.63	0.19	–

**Relative stiffness change**			

Predicted *K* at 38°C (Pa)	231.4	75.9	–
Relative slope (slope/predicted *K* at 38°C) (%/°C)	−3.89	−3.69	–

This table relates to Fig. 5. Regression analysis was conducted on all animals’ median *K* values (see Fig. 5, *gray dots*). A linear regression model was compared with a horizontal line, i.e., the assumption that temperature did not affect tissue elasticity, with an extra-sum-of-squares F-test. Despite gray and white matter elasticity in themselves being temperature dependent, the gray-to-white-matter elasticity ratio was not affected by temperature, as the relative elasticity changes in gray and white matter (normalized to the predicted *K* at body temperature, i.e., 38°C) were similar.

## Discussion

We have shown that the measured elasticity of spinal cord tissue strongly depends on the force and speed at which measurements are conducted. We confirmed that the AFM measurements themselves did not alter measured spinal cord elasticity to a biologically relevant degree within the explored parameter space (Fig. S2). To investigate the effect of directionality, we conducted our measurements in all three anatomical planes (transverse, horizontal, and sagittal; see Fig. 1, *F*–*H*). As force and speed increased, spinal cord stiffened in all tissue sections, in agreement with previous compressive tests in the CNS (10,14,15,20,21,30). However, we found region-specific differences in the tissue’s strain stiffening behavior: gray matter stiffened relatively less than white matter with increasing forces and speeds, so that the ratio of gray-to-white-matter elasticity *K*_*g*_*/K*_*w*_ decreased (Figs. 2 and S3). In one animal, *K*_*g*_*/K*_*w*_ fell below 1 for high forces and speeds (Fig. 3), but generally gray matter was stiffer than white matter within the explored parameter space. The distinct strain stiffening behavior of gray and white matter elasticity could be related to their different microarchitecture. Axons contain large amounts of intermediate filaments (neurofilaments), which show a very strong strain stiffening behavior if compared with other cytoskeletal components (31), potentially explaining why white matter stiffens relatively more than gray matter when exposed to larger strains.

Our experimental settings extended over two orders of magnitude, with forces ranging from 30 to 1500 nN and speeds ranging from 20 to 1200 *μ*m/s. With indenters of radius *R* = 44.65 *μ*m, this resulted in indentation depths of 4–40 *μ*m and indentation durations of 8–1278 ms in 95% of all measurements. Even though AFM settings do not directly translate into strains and strain rates, we estimate the nominal strain

(Equation 1) $$\varepsilon =\Delta l/l_{0}\approx \delta /h\text{,}$$

where Δl is the length change, $l_{0}$ the original length, δ the indentation depth (4–40 *μ*m), and h the sample height (999 *μ*m), to be between ∼0.4–4%, and the strain rate

(Equation 2) $$\dot{\varepsilon }=\frac{1}{2t_{\delta }}\text{,}$$

where $t_{\delta }$ is the indentation duration (8–1278 ms), to be approximately 0.4–60 Hz. Of note, local strains directly below the indenter will have been higher as the strain field is not uniform.

Both nanoindentation testing and MRE studies often find white matter to be stiffer than gray matter (Table 4). In nanoindentation measurements, indentations of between ∼5 and 10% of the sample height are commonly reported (14,15,32), sometimes even up to 20% (33), at speeds similar to the lower end of our parameter space (5–100 *μ*m/s (14,15,16,34)). MRE, on the other hand, exerts deformations of about 1–40 *μ*m in a large sample like a human brain (35,36,37), resulting in smaller strains than used in this study, but at higher strain rates of about 50–100 Hz (35,36,37,38,39,40). In our study, mean *K*_*g*_*/K*_*w*_ values approached 1 for high forces and speeds in the horizontal and sagittal planes, and we even observed *K*_*g*_*/K*_*w*_ falling below 1 in one animal (Fig. 3). This suggests that a *K*_*g*_*/K*_*w*_ < 1 as observed with nanoindentation and MRE could at least partially be the result of the even higher forces or speeds employed by these methods (Table 4). In addition, the much larger probe size of nanoindenters on the order of 750–1000 *μ*m radius (14,15,16,32,33,34) could lead to measuring collective behaviors of axon bundles at larger scales. Also, CNS exhibits both viscoelastic behavior (because of rearrangements of cells and the extracellular matrix) and poroelastic behavior (because of fluid flow through the matrix) (41). Differences in water displacement in gray compared with white matter could result in white matter appearing stiffer during shear wave propagation in MRE.

**Table 4:** Comparison of controversial data concerning gray and white matter mechanical properties in studies employing different strains and strain rates

**Publication** (citation)	**Conclusion** (gray-to-white matter ratio)	**Analysis model** (citation)	**Force***F*, I**ndentation***δ*, A**mplitude***a*	**Indenter** geometry and size (*μ*m)	**Nominalstrain***ε* (%)	**Strain rate**$\dot{\varepsilon }$, F**requency***f*, S**peed***s*
**Atomic force microscopy**						

This study	W < G (4.91) to W ≈ G (1.02)	Sneddon (28)	*F* = 30–1500 nN (*δ* = 4–40 *μ*m)	r_sphere_ = 44.65	∼0.4–4	*s* = 20–1200 *μ*m/s (*f* = 0.4–60 Hz)
Christ et al. (10)	W < G (1.54)	Hertz (27)	*δ* = 3 *μ*m (*F* ≤ 27.5 nN)	r_sphere_ = 18.64	1.30	*s* = 15 *μ*m/s
Moeendarbary et al. (12)	W < G (2.37)	Hertz (27)	*F* = 20–30 nN (*δ*_mean_ = 4 *μ*m)	r_sphere_ = 44.65	0.8	*s* = 5–10 *μ*m/s
Möllmert et al. (13)	W < G (1.75)	Sneddon (28)	*F* = 4 nN	r_sphere_ = 18.65	–	*s* = 10 *μ*m/s

**Nanoindentation**						

Dommelen et al. (14)	G < W (0.72)	Lee and Radok (54)	*δ* = 100 *μ*m; (*F* = 100–200 *μ*N)	r_sphere_ = 1000 *μ*m	5–10	*s* = 100 *μ*m/s
Budday et al. (15)	G < W (0.73)	Oliver and Pharr (55)	*δ* = 320 ± 20 *μ*m	r_circular flat punch_ = 750 *μ*m	6.8	*s* = 5 *μ*m/s
Weickenmeier et al. (16)	G < W (0.51)	Oliver and Pharr (55)	*δ* = 100 ± 10 *μ*m	r_circular flat punch_ = 750 *μ*m	2	*s* = 5 *μ*m/s

**Magnetic resonance elastography**						

Kruse et al. (35)	G < W (0.38)		*a* = 40 *μ*m			*f* = 100 Hz
Johnson et al. (36)	G < W (0.74)		*a* ≲ 5 *μ*m			*f* = 50 Hz
Clayton et al. (37)	G < W (various)		*a* ≈ 1 *μ*m			*f* = 45, 60, 80 Hz

Selected publications sorted by methodology. Strain-related parameters: force *F*, indentation *δ*, amplitude *a*. The controlled variable is indicated first. The resulting variable is indicated second in brackets, if such data are available. Nominal strain *ε* was calculated based on Equation 1 if sufficient data were available in the respective publication. Strain rate-related parameters: frequency *f*, speed *s.* G, gray matter; W, white matter.

*K*_*g*_*/K*_*w*_ was significantly higher in the transverse plane (perpendicular to the main direction of axonal fiber tracts) than in either the horizontal or the sagittal planes (both parallel to the main axonal fiber direction) (Fig. S7). In both longitudinal planes, *K*_*g*_*/K*_*w*_ values were similar, and closer to 1. Thus, measurement directionality relative to the predominant orientation of white matter tracts strongly affected *K*_*g*_*/K*_*w*_. We found gray matter to be isotropic with all parameter combinations and white matter to be transversely isotropic with some parameter combinations but anisotropic if comparing longitudinal and transverse directions, which agrees with previous findings from AFM and MRE studies (11,42). Overall, both gray and white matter elasticity decreased over time postmortem. Gray matter elasticity was initially stable for ∼3 h. It then decreased, before stabilizing again at ∼6 h postmortem at a lower level. In contrast, white matter displayed a pronounced drop of tissue elasticity in the first 6 h postmortem. Consequentially, the gray-to-white-matter elasticity ratio increased initially before plateauing after approximately 6 h postmortem (Fig. 4). Some previous studies reported brain tissue elasticity not to change for up to 5 days postmortem (15) or to start increasing ∼6 h after an initial stable phase (22). Tissues in these studies had been sourced from an abattoir, so it is possible that there were delays between death and brain tissue harvest. Furthermore, brains were stored in PBS, which cannot mitigate the complex metabolic changes affecting CNS tissue postmortem (26). It is therefore possible that the tissues used in these studies may have undergone mechanical changes already before the start of measurements, possibly even reaching a plateau phase as seen in the later time points of our data in both gray and white matter (Fig. 4, *A* and *B*).

MRE studies measuring mechanical brain tissue properties in vivo and again within ∼30–60 min postmortem in situ reported a considerable increase in tissue stiffness (23,43,44,45). This has been attributed to cytotoxic edema and brain swelling inside the spatially confined cranial cavity (23). If the brain was promptly taken out of the skull after death, the measured tissue elasticity was, in contrast, considerably lower than in vivo, which might be due to a sudden lack of blood and cerebrospinal fluid pressure (24). The studies agree that, after these immediate postmortem events, the measured tissue stiffness remains constant in the early postmortem phase until ∼1–3 h postmortem (23,24,44), compatible with our findings in gray matter (Fig. 4 *A*). At later time points, tissue stiffness was reduced (43,44), which further agrees with our findings in both gray and white matter.

We here identified clear differences in the susceptibility of gray and white matter elasticity for the passing of time. Using buffer solutions specifically optimized to ensure spinal cord slice viability and normal electrical activity of gray matter neurons for hours after slice preparation (26) likely helped slowing down the decay of the gray matter. In contrast, severing CNS axons in vivo leads to progressive axonal swelling, structural defects of the axonal cytoskeleton and the myelin sheath, and the accumulation of vacuoles within the first 6 h (46), potentially explaining the rapid decrease in white matter elasticity postmortem.

We confirmed temperature to be another important parameter impacting measured tissue elasticity. Tissue elasticity linearly increased as temperature decreased from physiological body temperature (38°C) to room temperature (20°C), in line with previously published results (24,25,47,48) (Fig. 5, *A* and *B*). Here, we found that the relative elasticity increase was similar for gray and white matter, and thus the gray-to-white-matter elasticity ratio was unaffected by temperature (Fig. 5 *C*). The liquid-crystal to gel-phase transition of myelin takes place at 63°C (49), so that considerable differences in the mechanical behavior of gray and white matter would only be expected above this threshold. This rules out that different measurement temperatures in past studies are responsible for the differences in the reported *K*_*g*_*/K*_*w*_ values.

### Limitations

While we have attempted to address some major contributors to reported differences in the gray-to-white-matter elasticity ratio, other factors might still play a role. Due to the scarcity of data available for the spinal cord specifically, we have compared our findings with data obtained on CNS tissue in general, i.e., both brain and spinal cord tissue. Both form a functional unit and contain gray and white matter. However, local mechanical differences exist even within gray and white matter (42,50), so that this division, while practically useful, remains somewhat crude. More work is needed to better understand the spatial heterogeneity in different brain and spinal cord regions and white matter tracts.

The anatomical context in which CNS tissue is examined is also important. While MRE allows for in vivo measurements, many other methods require postmortem tissue. As white matter is under tension (51,52), and this tension may be released during sample preparation, ex situ/in vitro measurements may not fully recapitulate in vivo/in situ mechanics. Furthermore, it is important to note that our AFM measurements constitute compressive testing and that measurements in tension or in shear could yield different results.

The fact that different methods use different models for data analysis and report different outcome variables poses a general problem for literature comparisons in the field and likely also contributes to inconsistent findings. The standard models to analyze AFM data are the Hertz and the Sneddon model. The Hertz model (27) is simpler and considered appropriate for low indentation depth relative to the indenter size, whereas the Sneddon model (28) has to be solved numerically but is accurate also at higher indentation depths. Here, we have used the Sneddon model for experiments examining the effect of different force-speed combinations, due to the greater indentation depths, and the Hertz model for all other experiments. Both models assume the sample to be isotropic, homogeneous, and linearly elastic. None of these conditions are met entirely by biological samples in the real world, but we designed our experiments to account for these violations. Samples were taken from specified anatomical locations and orientations to account for the lack of isotropy, and measurements were conducted in a spatially resolved way with a specified indenter size to account for the lack of homogeneity. We were working in a low-strain regime (*F* = 30 nN with the given indenter size), where the sample can be assumed close to linearly elastic. Despite exerting much higher forces in parts of this study, the Sneddon model still fit the force-indentation data curves well (Fig. 1, *B–E*). We also confirmed that the apparent reduced elastic modulus *K*, which we obtained with our analysis, was highly correlated with another commonly used – model-independent – measure, the stiffness *k* (unit: N/m), which we obtained by fitting a linear function to the force-indentation data in the range of 90–100% of the exerted force (Fig. S9; Pearson’s *r* = 0.95). However, other physical models can also take viscoelastic or poroviscoelastic effects into consideration, which would affect absolute measured values and possibly also *K*_*g*_*/K*_*w*_. Conducting creep or oscillatory measurements with AFM to assess viscoelastic effects in a spatially resolved way would be a great future asset for the community.

## Conclusions

Force, speed, and postmortem interval all influenced the gray-to-white-matter elasticity ratio *K*_*g*_*/K*_*w*_. Our results therefore reconcile seemingly contradictory findings in the literature concerning the relative elasticity of gray and white matter obtained with methods employing very different strain and strain rate regimes. Given the tissue compartment-specific dependence of elasticity on these parameters, it is important to tune the choice of method to the question being asked. If the mechanical environment experienced by cells in vivo is in focus, small strains and strain rates should be used. If data are to be acquired for modeling of high-impact scenarios such as traffic accidents, high strains and strain rates should be chosen to learn how the tissue behaves under these conditions.

Furthermore, high-quality and timely sample preparation is paramount for reliable measurements of CNS tissue elasticity, in particular if white matter is concerned. Attention also must be paid to the method chosen for euthanasia, which can impact mechanical tissue properties (53), and immediate supply of (i.e., perfusion with) a physiological buffer, such as artificial cerebrospinal fluid solutions (26), must be ensured. Careful consideration of sample preparation techniques and selection of measurement parameters, which mimic the relevant in vivo situation more closely, will lead to more impactful results, as well as better standardization and comparability of results across techniques and laboratories.

Our findings therefore suggest that the contradictory findings in the field might at least partly be due to different strain and strain rate regimes employed by different measurement methods, as well as testing being conducted along different anatomical axes, and illustrate the complex nonlinear nature of CNS tissue mechanics. Future research should investigate if further parameters such as probe size and poroviscoelastic properties of CNS tissue also contribute to the apparently contradictory *K*_*g*_*/K*_*w*_ values reported in the literature.

## Acknowledgments

The authors thank the animal facility staff for their excellent work and the research groups that donated animals to this project. This work was supported by the 10.13039/100010269Wellcome Trust (PhD fellowship 211614/Z/18/Z to J.M.B.), the 10.13039/501100000781European Research Council (Consolidator Award 772426 and Synergy Grant 101118729 to K.F.), the 10.13039/501100001659German Research Foundation (DFG) (projects in 460333672 CRC1540 EBM and 270949263 GRK2162 to K.F.), and the 10.13039/100005156Alexander von Humboldt Foundation (Alexander von Humboldt Professorship to K.F.).

## Author contributions

J.M.B., E.K., and K.F. designed the study. J.M.B. performed the experiments and analyzed the data. A.K.W. provided technical support and contributed software. J.M.B. and K.F. wrote the manuscript, with contributions from all coauthors.

## Declaration of interests

The authors declare no competing interests.

## Supporting material

Document S1. Figures S1–S9

Table S1. Number of animals (*N*) and AFM measurements (*n*) contributing to gray and white matter measurements at different force-speed combinations in all three anatomical planes For every force-speed combination investigated in each anatomical plane in both gray and white matter shown in Fig. 2, this table provides the numbers of animals (*N*), the total number of measurement points (*n*_total_), the minimum and maximum number of measurement points per animal (*n*_min_/*N* and *n*_max_/*N*), the animal IDs, the median reduced apparent elastic modulus per animal, and the mean and standard deviation of the median reduced apparent elastic moduli across all investigated animals. The latter two measures have been used for data representation in Figs. 2, S3, and S4. In total, 27,940 AFM measurements contributed to this data set. Raw data are available online (Zenodo: https://doi.org/10.5281/zenodo.14630529).

Table S2. Number of animals (*N*) contributing to the calculation of the gray-to-white-matter elasticity ratio *K*_g_/*K*_w_ at different force-speed combinations in all three anatomical planes For every force-speed combination investigated in each anatomical plane shown in Fig. 2, this table provides the numbers of animals (*N*), the animal IDs, the *K*_g_/*K*_w_ ratio for each animal (calculated by dividing the median gray matter reduced apparent elastic modulus by the median white matter reduced apparent elastic modulus), and the mean and standard deviation of the *K*_g_/*K*_w_ ratios across all investigated animals. The latter two measures have been used for data representation in Figs. 2, S3, and S4. Raw data are available online (Zenodo: https://doi.org/10.5281/zenodo.14630529).

Document S2. Article plus supporting material
