# Supplementary material for: Measurement force, speed, and postmortem time affect the ratio of CNS gray-to-white-matter elasticity
Source: Biophys J. 2025 Mar 16;124(24):4485–96. doi: 10.1016/j.bpj.2025.03.009 (PMC12821018; doi:10.1016/j.bpj.2025.03.009)
Supplement: Document S1. Figures S1–S9 [file mmc1.pdf]

Biophysical Journal, Volume *124*

Supplemental information

Measurement force, speed, and postmortem time affect the ratio of CNS gray-to-white-matter elasticity

Julia Monika Becker, Alexander Kevin Winkel, Eva Kreysing, and Kristian Franze

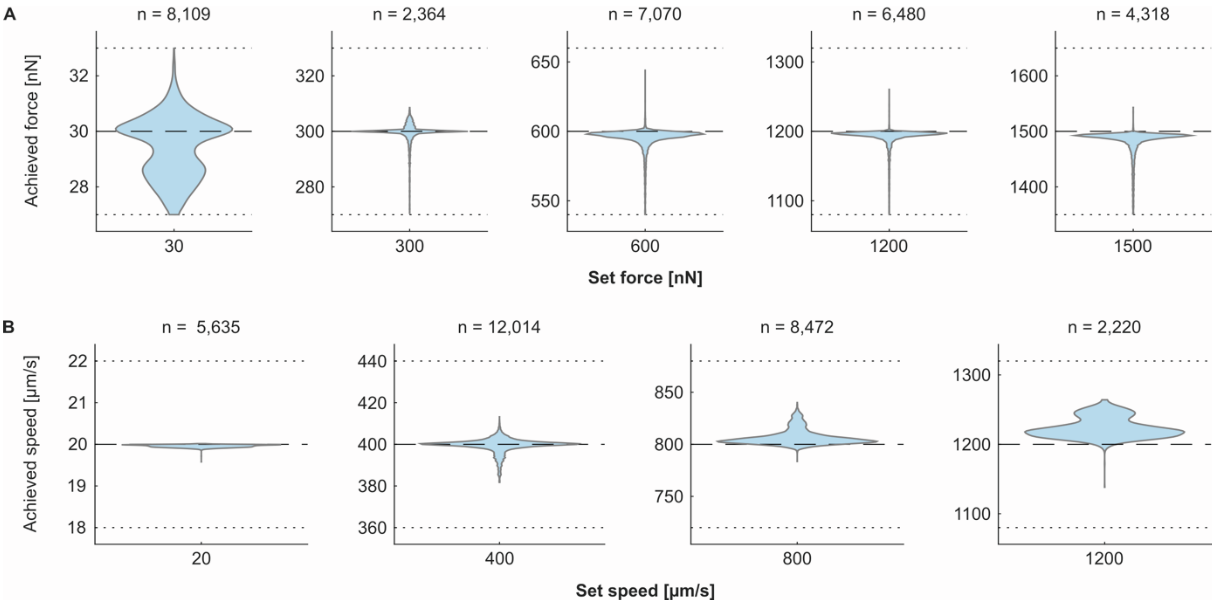

Figure S1: AFM accurately achieves setpoint forces and speeds across a wide range. **(A)** Actually achieved forces and **(B)** speeds plotted against setpoint (= target) forces and speeds. The target forces and speeds are indicated by dashed lines, ±10% of target forces and speeds are indicated by dotted lines. The number of measurements analysed is indicated above each plot. Measurements for which the achieved force deviated by more than 10% from the target force were excluded from further data analysis. No achieved speed exceeded the target speeds by more than ±10%.

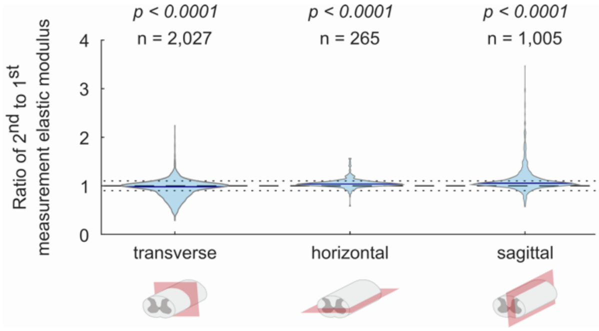

Figure S2: Measurements conducted in the chosen parameter space do not alter the tissue's apparent elastic modulus to a relevant degree. Data relate to Figure 2. To assess whether AFM measurements with the highest forces and speeds employed there mechanically damage the tissue, AFM measurements were taken with 30 force-speed combinations at the same location and were then repeated once. Apparent elastic moduli of the second measurements were compared to the first ones. Data shown are pooled for each anatomical plane and were compared against a value of 1 with a two-tailed one-sample t-test. The number of measurements per plane (*n*) and the p-values are indicated in the figure. The distribution medians were close to 1 (transverse: 0.981; horizontal: 1.037; sagittal: 1.053; all indicated with blue lines), indicating that no experimentally meaningful mechanical alterations had occurred. The dashed line indicates a ratio of 1, the dotted lines denote 1±10%.

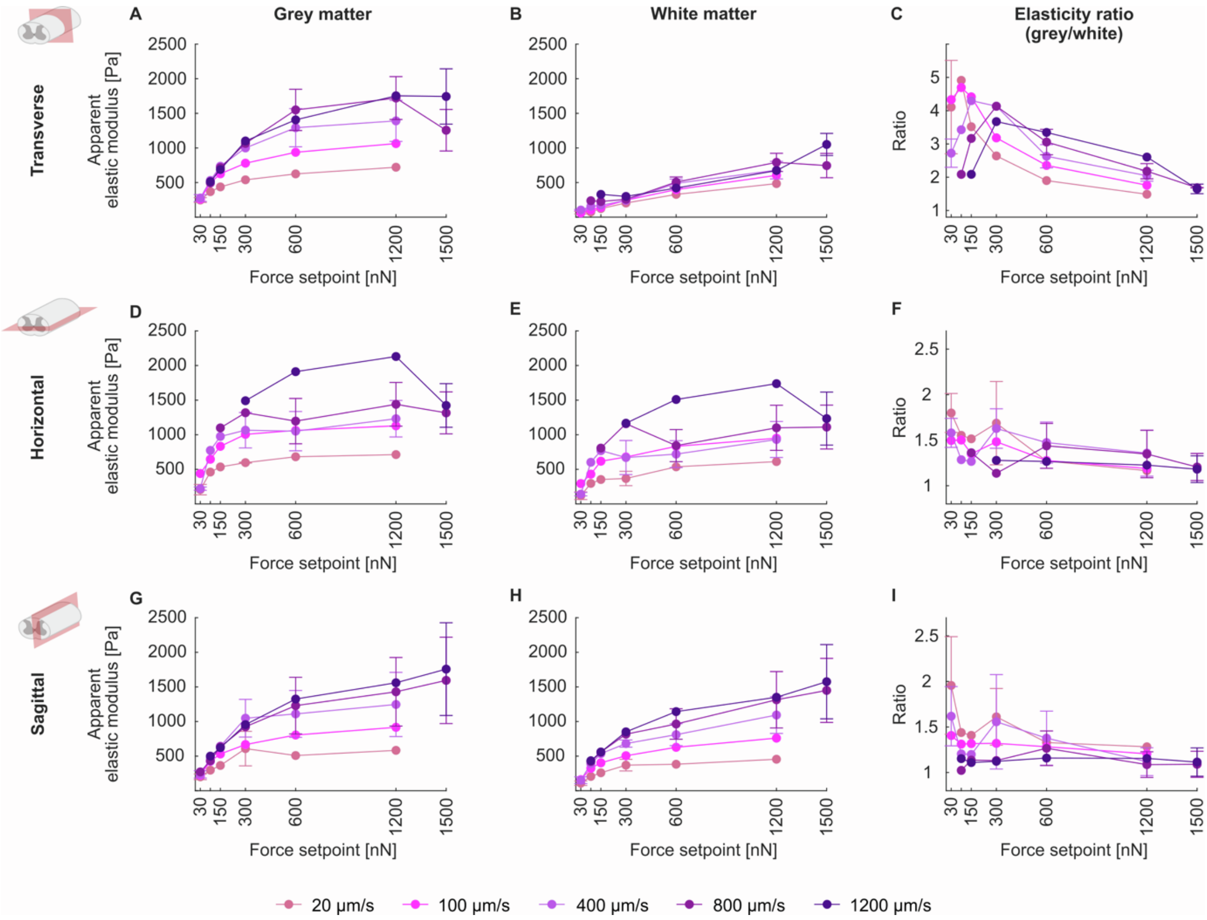

Figure S3: Increasing the force setpoint generally increases grey and white matter elasticity and decreases the grey-to-white-matter elasticity ratio. Projections of data shown in Figure 2, showing measured elasticity versus force. Dots and error bars represent means and standard deviations of all animals' median *K* (**A, B, D, E, G, H**) or K_g/K_w ratios (**C, F, I**). Where error bars are missing, N = 1 animal. For details about animal numbers, measurement numbers and median elasticity values and ratios from individual animals, please refer to Tables S1 and S2. Data are colour-coded according to the setpoint speed at which they were acquired; data acquired at the same speed are connected by lines.

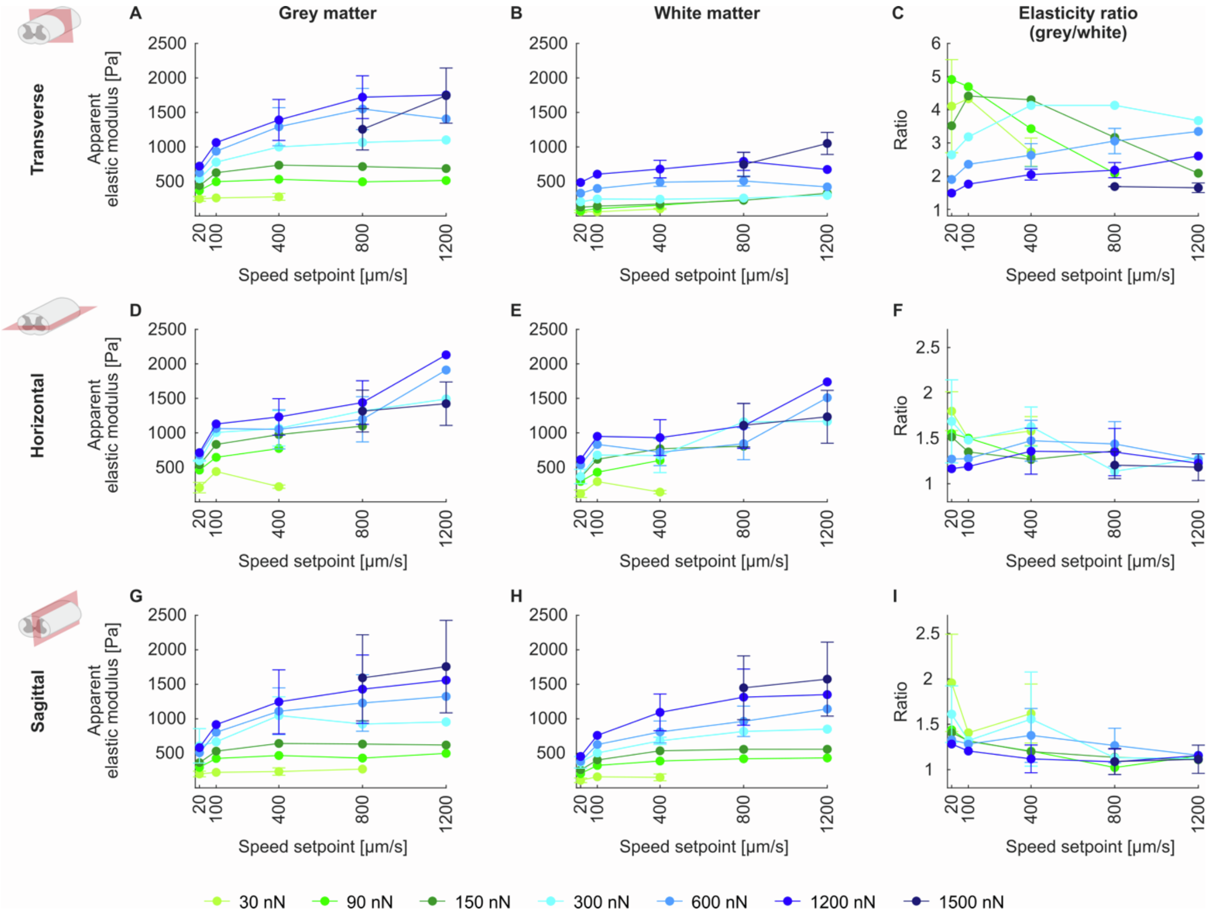

Figure S4: Increasing the speed setpoint generally increases grey and white matter elasticity and decreases the grey-to-white-matter elasticity ratio. Projections of data shown in Figure 2, showing measured elasticity versus speed. Dots and error bars represent means and standard deviations of all animals' median K (**A, B, D, E, G, H**) or K_g/K_w ratios (**C, F, I**). Where error bars are missing, N = 1 animal. For details about animal numbers, measurement numbers and median elasticity values and ratios from individual animals, please refer to tables S1 and S2. Data are colour-coded according to the setpoint force at which they were acquired; data acquired with the same force are connected by lines.

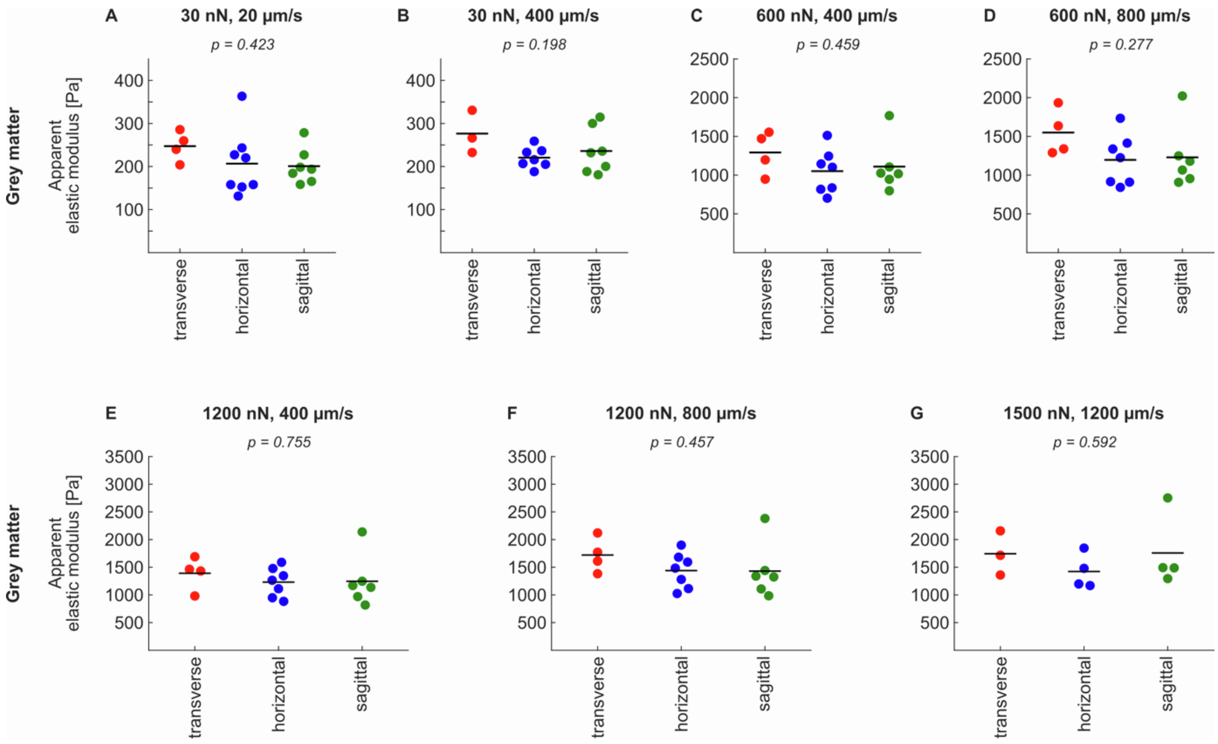

Figure S5: Grey matter elasticity does not significantly differ across the three anatomical planes. Data relate to Figure 2. (**A-G**) Comparison of elasticity values obtained with the same parameter combinations in the three anatomical planes. Dots represent individual animals' median *K*, bars their means. Anatomical planes were compared with ANOVA. None of the comparisons were significant ($p > 0.05$ in all cases). Parameter combinations were included in the analysis if data was available for all three anatomical planes and if $N \geq 3$ animals/plane ($N = 3 – 8$ animals/plane). Data shown here are included in Table S1.

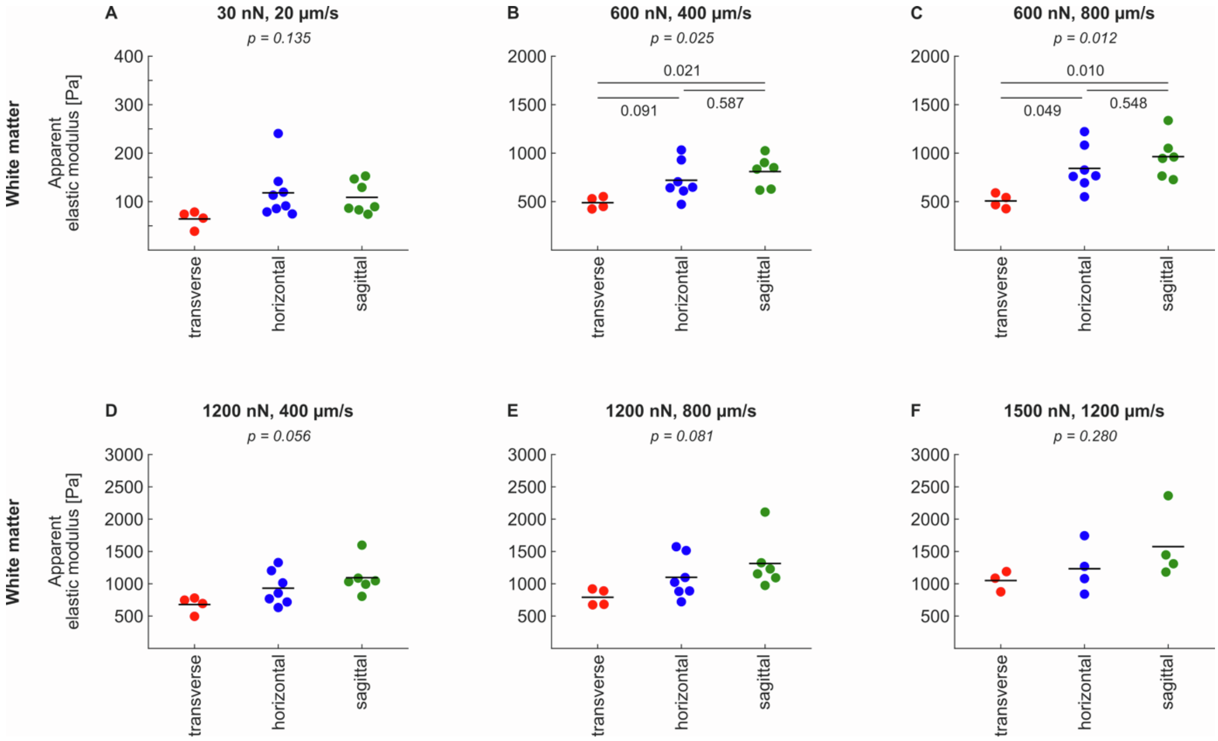

Figure S6: White matter elasticity is lowest in the transverse plane. Data relate to Figure 2. (**A-F**) Comparison of elasticity values obtained with the same parameter combinations in the three anatomical planes. Dots represent individual animals' median *K*, bars their means. Anatomical planes were compared with ANOVA (p-values in italics at the top of each panel). Where ANOVA yielded $p \leq 0.05$ (**B, C**), Tukey's multiple comparisons test was used to compare individual planes (comparisons indicated by bars with adjacent p-values). Parameter combinations were included in the analysis if data was available for all three anatomical planes and if $N \geq 3$ animals/plane ($N = 3 - 8$ animals/plane). Data shown here are included in Table S1.

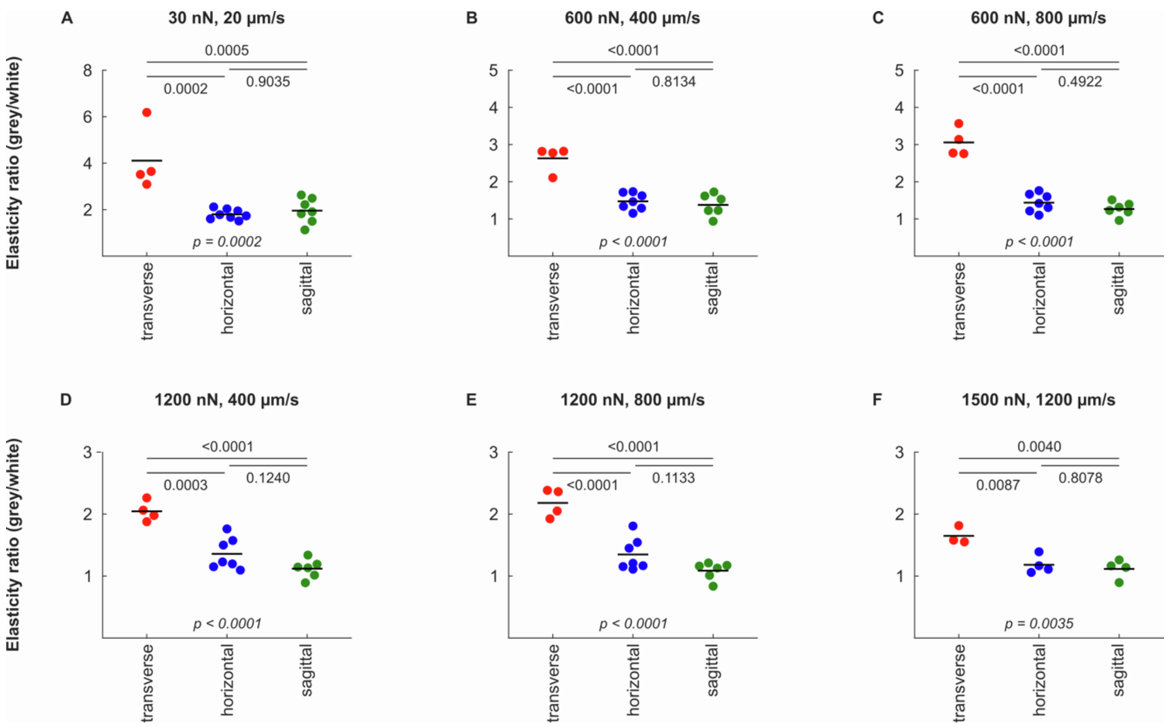

Figure S7: Measurement directionality significantly affects the K_g/K_w ratio. Data relate to Figure 2. (**A-F**) Comparison of K_g/K_w ratios obtained with the same parameter combinations in the three anatomical planes. Dots represent individual animals' K_g/K_w ratios, bars their means. Anatomical planes were compared with ANOVA (p-values in italics at the bottom of each panel; all significant), followed by Tukey's multiple comparisons test (indicated by bars with adjacent p-values). In all cases, K_g/K_w values were significantly higher in the transverse plane than in either the horizontal or sagittal planes. In all cases, K_g/K_w ratios obtained in the horizontal and the sagittal plane did not significantly differ. Parameter combinations were included in the analysis if data was available for all three anatomical planes and if $N \geq 3$ animals/plane ($N = 3 – 8$ animals/plane). Data shown here are included in Table S2.

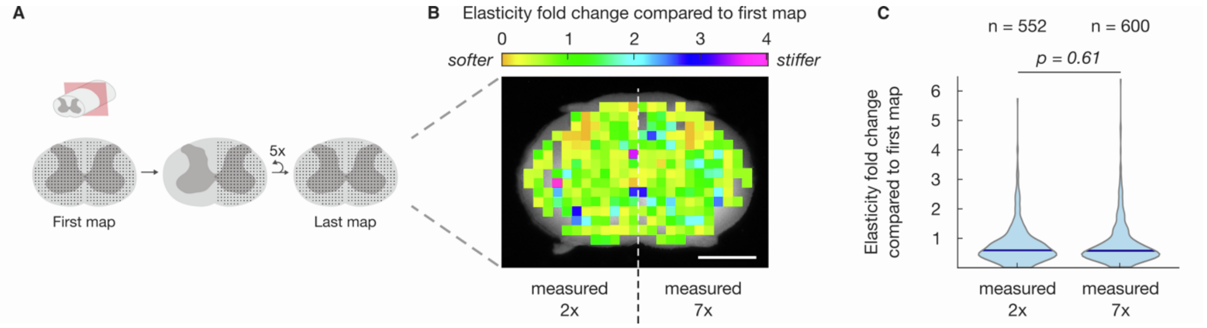

Figure S8: Multiple repeated low-force-low-speed measurements do not alter the apparent elastic modulus of spinal cord tissue. (**A**) Initially, an AFM map was measured on each half of a spinal cord cross-section. Subsequently, only one of the two grids was measured five more times every ~30 or 60 minutes. Finally, both sides were measured again. The initial maps were acquired between 1:38 h and 3:51 h (mean: 2:49 h) post-mortem, measurements in the last maps between 8:31 h and 11:06 h (mean: 9:45 h) post-mortem. (**B**) Relative elasticity changes between the first and last AFM map in both sides of the spinal cord cross-section in a representative animal. The heatmap illustrates midline symmetry (midline annotated with a dashed line). The left side was measured twice, whereas the right side of the spinal cord was measured seven times within the same time interval. (**C**) Distribution of elasticity changes between the first and last AFM maps in areas measured twice and areas measured 7 times (97 – 126 measurements per map per animal, N = 5 animals). Both conditions were compared with a two-tailed unpaired t-test after log-transformation ($p = 0.61$). The five additional AFM elasticity mapping experiments did not significantly change the elasticity of the tissue. Median for “measured 2x” is 0.595; median for “measured 7x” is 0.582. Scale bar = 1000 µm.

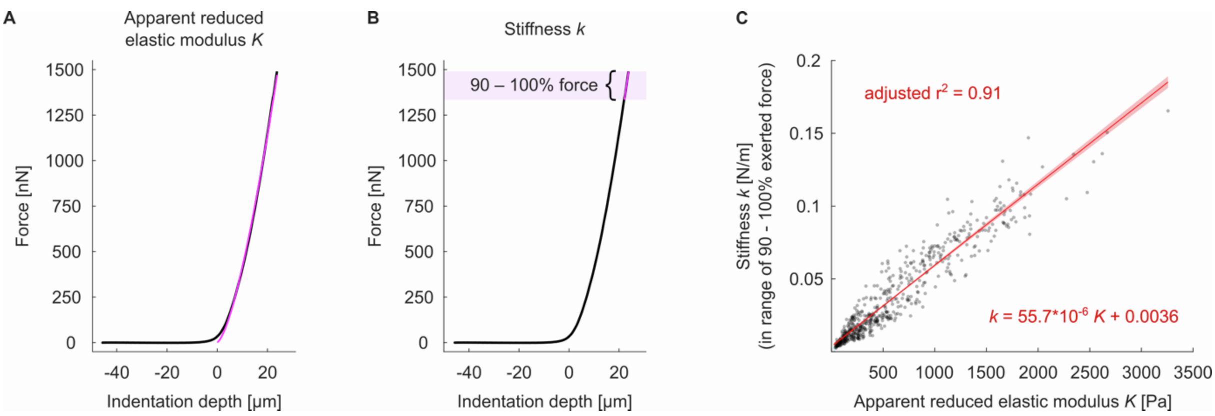

Figure S9: Apparent reduced elastic modulus *K* and stiffness *k* are tightly correlated. Data relate to Figures 1 and 2. AFM force-distance curves were fitted with (**A**) the Sneddon model (pink line) to obtain the apparent reduced elastic modulus *K*, and (**B**) a linear function (pink line) fitted to the range of 90 – 100% of the exerted force (area shaded in light pink) to obtain the stiffness *k* (i.e. the slope of the function). (**A**) and (**B**) show the same example curve. (**C**) Correlation between the apparent reduced elastic moduli *K* and stiffnesses *k*. We found a high degree of positive correlation (Pearson's correlation coefficient: 0.95), suggesting that both approaches obtain qualitatively similar results. Grey dots represent individual force-distance curves (n = 559 force-distance curves, representing all force-speed combinations from all three anatomical planes; if >10 curves per combination existed, 10 curves were sampled randomly). Red line with shaded red area: Linear regression with 95% confidence interval. Regression parameters and adjusted r^2 value are indicated in the figure panel.

Table S1: Number of animals (N) and AFM measurements (n) contributing to grey and white matter measurements at different force-speed combinations in all three anatomical planes.

For every force-speed combination investigated in each anatomical plane in both grey and white matter shown in Figure 2, this table provides the numbers of animals (N), the total number of measurement points (n_{total}), the minimum and maximum number of measurement points per animal (n_{min}/N and n_{max}/N), the animal IDs, the median reduced apparent elastic modulus per animal, and the mean and standard deviation of the median reduced apparent elastic moduli across all investigated animals. The latter two measures have been used for data representation in Figure 2 and Figures S3 and S4. In total, 27,940 AFM measurements contributed to this dataset. Raw data are available online (Zenodo: https://doi.org/10.5281/zenodo.14630529).

Table S2: Number of animals (N) contributing to the calculation of the grey-to-white-matter elasticity ratio K_g/K_w at different force-speed combinations in all three anatomical planes.

For every force-speed combination investigated in each anatomical plane shown in Figure 2, this table provides the numbers of animals (N), the animal IDs, the K_g/K_w ratio for each animal (calculated by dividing the median grey matter reduced apparent elastic modulus by the median white matter reduced apparent elastic modulus), and the mean and standard deviation of the K_g/K_w ratios across all investigated animals. The latter two measures have been used for data representation in Figure 2 and Figures S3 and S4. Raw data are available online (Zenodo: https://doi.org/10.5281/zenodo.14630529).
